# Strategy for pacemaker implantation following transcatheter tricuspid valve replacement: A real world single centre experience

**DOI:** 10.1007/s10840-025-02168-8

**Published:** 2025-12-04

**Authors:** L. Friedrich, M. Walgenbach, M. O`Connor, A. Fetcu, F. Geißenberger, F. Haug, C. Pellegrini, T. Rheude, H. A. Alvarez-Covarrubias, E. Xhepa, T. Trenkwalder, S. Voss, M. Krane, H. Schunkert, H. Ruge, M. Joner, C. Lennerz

**Affiliations:** 1 Department of Cardiovascular Diseases, German Heart Center, School of Medicine and Health, TUM University Hospital, Technical University of Munich, Germany, TUM University Hospital, Technical University of Munich, Lazarettstraße 36, 80636 München, Munich, Germany; 2 Department of Cardiovascular Surgery, German Heart Center, School of Medicine and Health, TUM University Hospital, Technical University of Munich, Germany , Munich, Germany; 3 Department of Electrophysiology, Te Toka Tumai Auckland City Hospital, Auckland, New Zealand; 4 DZHK (German Centre for Cardiovascular Research), partner site Munich Heart Alliance, Munich, Germany; 5 Hospital de Cardiología, Centro Médico Nacional Siglo XXI, IMSS, Ciudad de México, Mexico

**Keywords:** Tricuspid valve, Transcatheter tricuspid valve replacement, Cardiac implantable electronic device, Leadless pacemaker, Coronary sinus lead, Valve sparing pacemaker

## Abstract

**Background:**

High-degree AV-Block requiring pacemaker (PM) implantation is a frequent complication after transcatheter tricuspid valve replacement (TTVR), which represents a relative contra-indication to standard right ventricular (RV) lead placement. We report our experience in this patient cohort and compared different PM strategies prioritising interventional valve sparing options.

**Methods:**

Consecutive patients treated with the EVOQUE system (Edwards Lifesciences) at our centre between January 2024 and May 2025 were included in this retrospective single centre analysis. Successful implantation of a valve sparing PM and feasibility of PM implantation were assessed.

**Results:**

This analysis included 39 patients (mean age 77.3 years, 74.4% women, 8 patients with pre-existing PM). High-degree AV-Block requiring PM implantation occurred in 28.2% in our cohort (11/39 patients), the de-novo PM implantation rate was 35.5% (11/31 patients). Complete or incomplete right bundle branch block (RBBB) was the most frequent conduction system disorder after TTVR (11/31 patients). Median time from TTVR to PM implantation was six days [IQR: 2.5–13]. PM implantation was feasible in 11/11 patients (100%), no surgical lead placement was necessary. Implantation of a valve sparing PM was successful in 10/11 patients (90.9%). Leadless PM were implanted in 3/11, coronary sinus lead PM in 7/11 patients. In one patient both interventional valve sparing PM approaches failed and a standard RV lead across the EVOQUE system was implanted.

**Conclusion:**

High-degree AV-Block after TTVR is a frequent complication and PM implantation in these patients demands careful consideration. Valve sparing PM approaches appear feasible and were achieved with reasonable success rates in our cohort.

**Graphical Abstract:**

**Figure:**
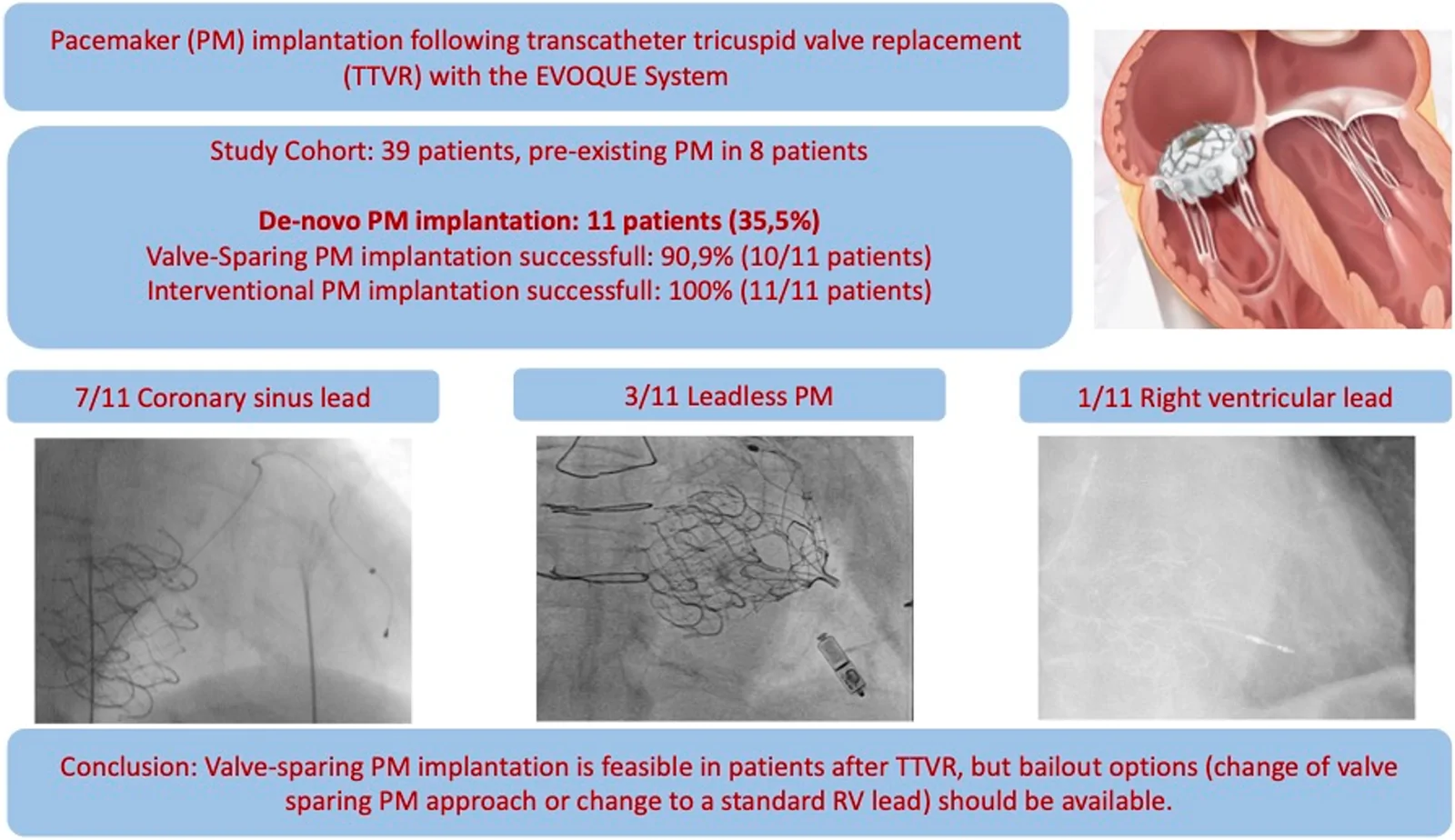

**Supplementary information:**

The online version contains supplementary material available at https://doi.org/10.1007/s10840-025-02168-8.

## Introduction

Recently the therapeutic landscape for severe tricuspid insufficiency has rapidly advanced, driven primarily by innovations in transcatheter treatment. Transcatheter annuloplasty (CARDIOBAND) [1], tricuspid transcatheter edge-to-edge repair (T-TEER) [2], heterotopic valve implantation (TRICVALVE) [3] and transcatheter tricuspid valve replacement (TTVR) have been introduced as possible treatment options [4]. The EVOQUE Transcatheter Tricuspid Valve Replacement System (Edwards Lifesciences), the first approved interventional tricuspid valve replacement system, demonstrated safety and efficacy in the TRISCEND trial [5]. In TRISCEND II, a randomized controlled trial with 400 patients (randomized 2:1), TTVR with the EVOQUE system demonstrated superiority to medical therapy in a primary composite outcome, driven by improvements in symptoms and quality of life [6].

Both studies reported pacemaker (PM) implantation as a common complication of this new procedure. In patients without a pre-existing PM, TRISCEND reported new PM implantation in 13.3% of patients at one year, TRISCEND II reported new PM implantation in 27.8% of patients at one year. First real-world evidence of 176 patients treated with the EVOQUE system showed a rate of PM implantation of 18.9% in PM naive patients after 30 days [7]. Vulnerable exposure of the His-Bundle and its branches along the membranous and the ventricular septum in combination with mechanical stress caused by oversizing of the EVOQUE system in relation to the annulus is hypothesised to be the relevant pathophysiological cause of this complication, drawing parallels to the experience with transcatheter aortic valve replacement (TAVR) [8, 9].

PM implantation following TTVR represents a challenge because of potential issues with right ventricular access for pacing leads. Experience in placing a standard right ventricular (RV) lead over the EVOQUE system is limited. While isolated successful implantations have been reported [6, 7], possible valve-lead interaction raises concern about mid- to long-term valve and lead function. In the case of PM implantation after surgical valve replacement sparing PM options are recommended [10], similarly transvalvular lead placement was avoided in the majority of published PM implantations after EVOQUE. Valve sparing PM options include leadless pacing and pacing via a coronary sinus (CS) lead, an established concept in cardiac resynchronisation therapy (CRT). Beside these pacing options, a surgical approach with implantation of epicardial leads remains another possible valve sparing back-up solution. The optimal treatment strategy for PM implantation after TTVR is unclear in terms of demand and timing of PM implantation as well as choice of pacing strategy. Against this background we report our first experience with different pacing strategies in patients requiring permanent PM implantation after TTVR with the EVOQUE system.

## Methods

### Patient population and characteristics

 We retrospectively reviewed patients undergoing TTVR with the EVOQUE system between January 2024 and May 2025 at the German Heart Center Munich (TUM University Hospital). The study was approved by the local ethics committee of the Technical University of Munich and was conducted according to the principles of the Declaration of Helsinki. The study was registered at clinicaltrials.gov with the identifier NCT06814574.

All consecutive patient undergoing TTVR with the EVOQUE system during the study period were included. Clinical information before and after TTVR was derived from our database and included baseline and procedural characteristics, ECG parameters, echocardiographic characteristics and outcome data.

### PM implantation and follow-up

The indication for PM implantation was determined in accordance with current ESC guidelines [10]. Following the guideline we define high grade AV-block in sinus rhythm as second-degree Mobitz typ II (2:1 ratio or higher) and third-degree AV-block. High grad AV-block was also defined as symptomatic bradycardia or ventricular escape rhythm in patients with atrial fibrillation. After TTVR we favour valve sparing solutions and therefore planned either coronary sinus lead pacing or leadless PM implantation. The choice between these options was at the discretion of the implanter. We document the initially determined implantation approach and register any change in implantation strategy. We defined success as the implantation of a valve sparing PM system. We define feasibility as implantation of any PM system without the need for surgical epicardial leads. This includes valve sparing system as well conventional PM with a transvalvular lead. Procedural data on PM implantation were collected including placement of a temporary PM, procedural time, fluoroscopy time, type of PM, crossover to an alternative implantation strategy, number and type of electrodes as well as standard electrical parameters. Every patient received one additional routine device interrogation with report of standard electrical parameters during the index hospitalization. Complications in connection with the PM implantation within in the index stay were recorded. Follow-up of patients without PM implantation consisted of in-hospital monitoring, planned visits and unplanned readmissions to our clinic.

### Statistical analysis

Statistical analysis was conducted using IBM SPSS Version 30. Continuous variables are presented as mean ± standard deviation (SD), follow-up time in days as median and interquartile range (IQR). Categorical variables are described as absolute numbers and percentages (n (%)). ECG characteristics and changes were tested as predictive factors for PM implantation using univariable logistic regression and Fisher’s exact test. Odds ratios (OR) and their 95% confidence intervals (CI) were calculated and reported, P-values < 0.05 were considered statistically significant.

## Results

### Patients and primary outcome

**Fig. 1:**
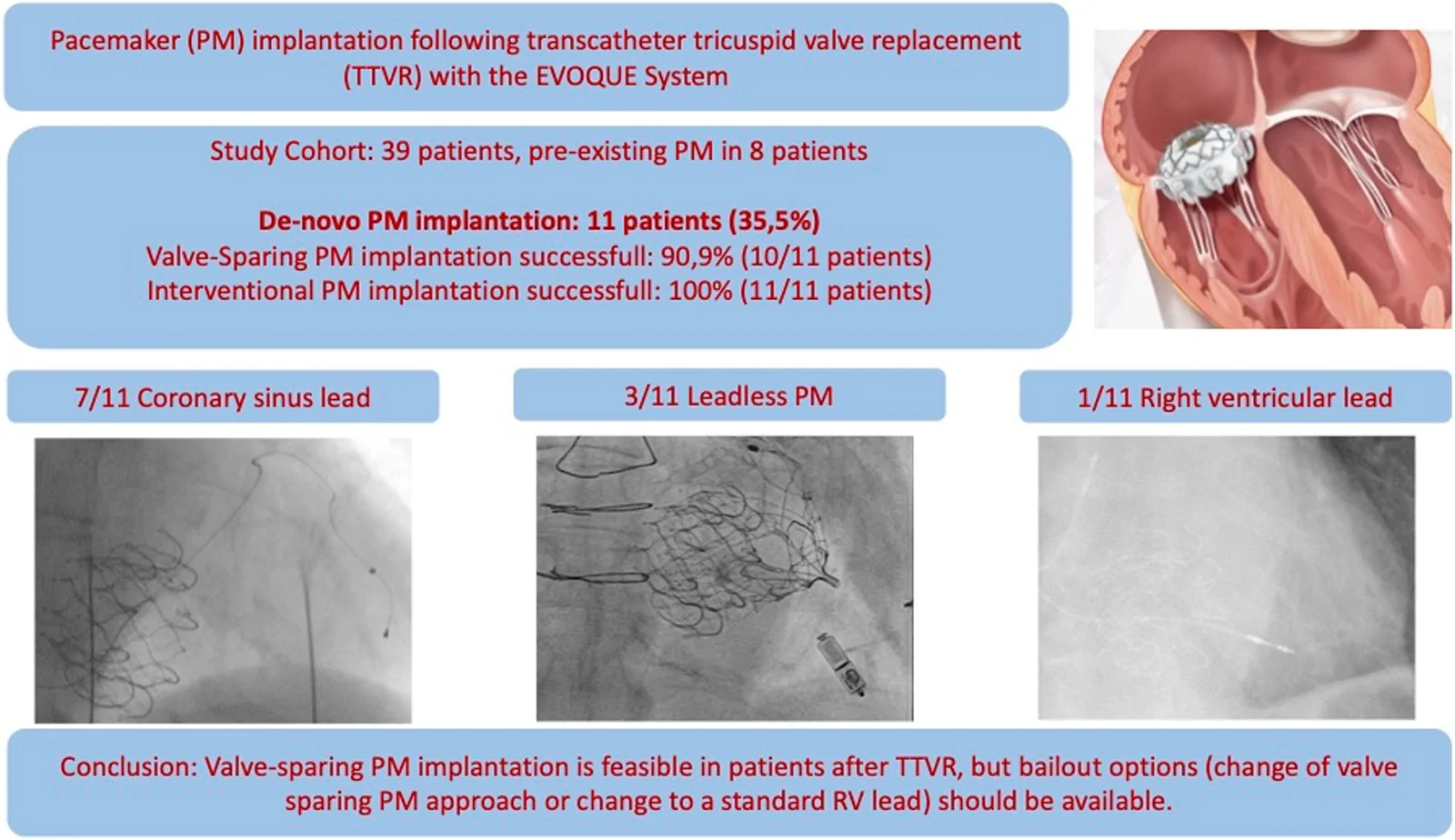
Illustration of the EVOQUE-System was provided by Edwards Lifesciences LLC, Irvine, CA. Edwards, Edwards Lifesciences, the stylized E logo, EVOQUE and EVOQUE Eos are trademarks of Edwards Lifesciences Corporation

Between January 2024 and May 2025, a total of 39 patients received TTVR with the EVOQUE system and were included in our analysis. TTVR was successful in all 39 patients. PM implantation was performed in 11 patients (28.2%). In our cohort, 8 patients (20.5%) had a pre-existing transvenous PM, resulting in a de-novo rate of permanent PM implantations of 35.5% (11/31 patients). Median follow-up for patients without pre-existing or new PM implantation was 46 days [IQR: 7–105 days]. Baseline characteristics of our cohort are shown in Table 1 and our primary results in Fig. 1.

**Table 1:** Baseline characteristics

	All Patients (*n* = 39)
Age (year, mean, SD)	77.3 ± 7.7
Gender (female, n, %)	29 (74.4)
Mead body mass index (mean, SD)	25.4 ± 5.5
Atrial fibrillation (n, %)	37 (94.9%)
Hypertension (n, %)	33 (84.6%)
Coronary heart disease (n, %)	21 (53.8%)
Chronic obstructive pulmonary disease (n, %)	3 (7.7%)
Liver disease (n, %)	6 (13.4%)
Diabetes mellitus (n, %)	12 (30.8%)
Previous stroke (n, %)	7 (17.9%)
Creatinin (mg/dl, mean, SD)	1.1 ± 0.40
Glomerular filtration rate (CKD-EPI; ml/min, mean, SD)	57.4 ± 19.7
Mean left ventricular ejection fraction (mean, SD)	53.5 ± 6.9
Previous heart surgery (n, %)	12 (30.8%)
Pre-existing pacemaker (n, %)	8 (20.5%)

**Fig. 2:**
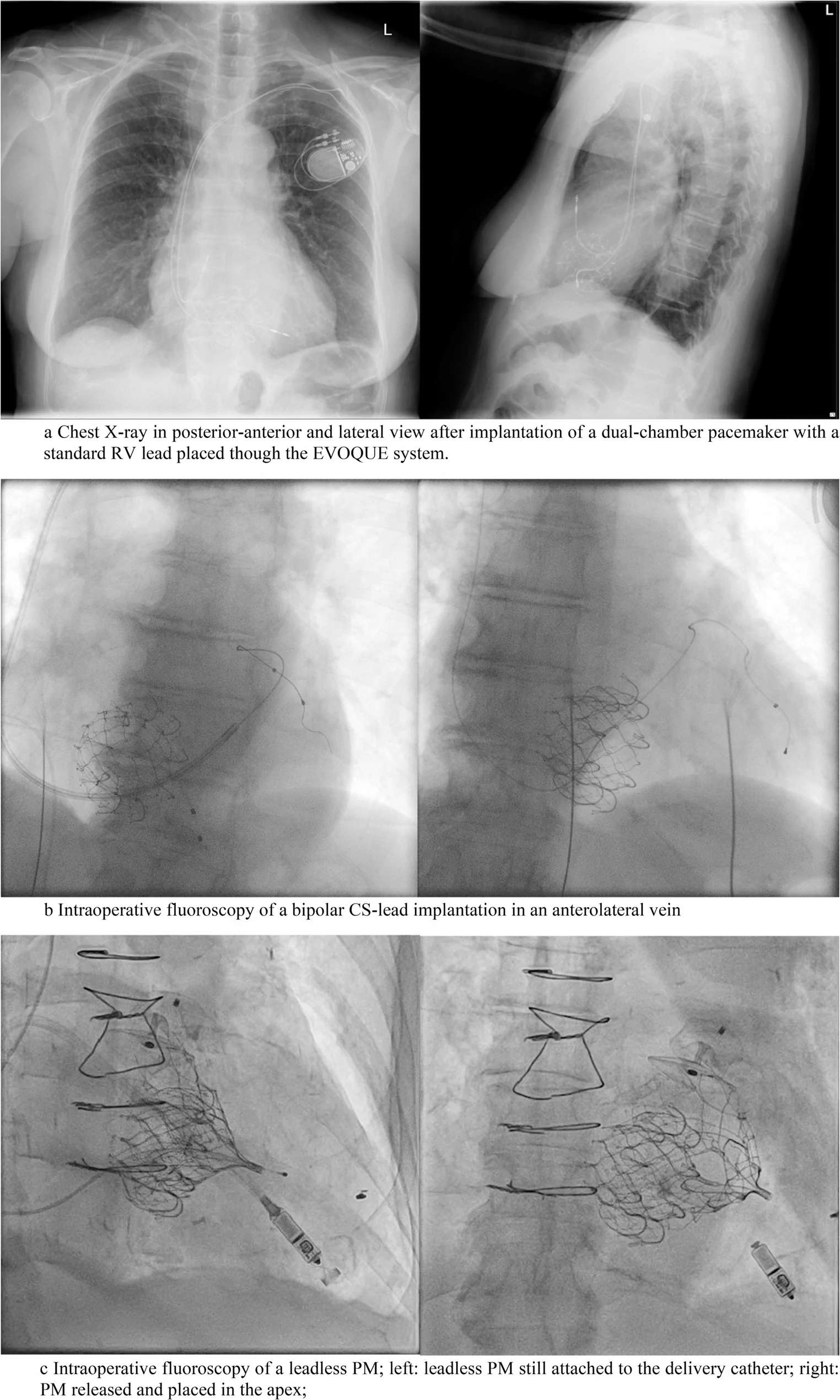
PM-systems post TTVR

A valve sparing approach was successful in 10/11 PM implantations resembling a success rate of 90.9%. In these ten patients, the a priori chosen pacing approach was successful. Three patients received a leadless PM and seven patients received a coronary sinus lead. Feasibility of implanting an PM was 100% (11/11), no surgical placement of epicardial leads was necessary. However, in one patient a valve sparing option could not be achieved. In this patient, the primary approach of a leadless pacemaker (Micra AV, Medtronic) was aborted because of inability to cross the prosthetic valve. The main limitation appeared to be steric, as the large delivery catheter had restricted manoeuvrability in the right atrium and was repeatedly obstructed at the valve frame. With a deep right atrial approach using a steerable sheath, mobility was markedly limited and the valve orifice plane could not be reached. A higher atrial approach provided better catheter mobility, but the angle was too steep to allow passage across the valve. Despite repeated attempts by two experienced operators, crossing the prosthetic valve was unsuccessful. The alternative approach of placing a coronary sinus lead was also aborted after placement of the CS lead in all accessible target vessels was unsuccessful because of an unstable position or unacceptable electrical parameters (high pacing thresholds, phrenic nerve stimulation). As a bailout strategy a standard RV lead was placed through the bioprosthetic valve without complications. Echocardiography post TTVR, post PM implantation and at a routine follow-up at 3 months revealed no change in valve function and reported a mild (°I) and stable valvular insufficiency. Figure 2a-c show the different PM approaches in intra/postoperative fluoroscopy.

### ECG parameters and timing of PM implantation

The majority of 8/11 implantations were carried out during the index hospitalization. The median time from TTVR to pacemaker implantation was six days [IQR: 2.5 – 13 days]. The remaining three patients were discharged and readmitted for PM implantation. PM implantation in these patients was carried out on day 15, 35 and 41 after the index procedure. One patient presented in an emergency setting in cardiogenic shock due to bradycardia, the second patient presented in the emergency department with aggravation of dyspnoea due to cardiac decompensation whereas the third patient mentioned dizziness in a routine follow-up visit and Holter monitoring revealed intermittent high-degree AV-block.

The indication for PM implantation was high-degree AV-block in all patients. ECG comparison in patients without prior implanted PM pre- and post-EVOQUE (n=31) revealed several differences. Most frequently new onset of RBBB or incomplete RBBB developed in 11 patients, 5 of which subsequently received a PM. Furthermore, two patients in sinus rhythm developed new onset AV-Block °I, both also subsequently received a PM. ECG parameters, indication and time to implantation for all patients are accessible in the supplementary material.

Frequent periinterventional ECG changes were analysed as predictors for permanent pacemaker implantation, including new bundle branch block (BBB), specifically new (complete) right bundle branch block (RBBB), QRS-prolongation>20ms and new-onset AV-block °I (AV-interval >200ms). Univariable logistic regression showed positive odds ratios indicating increased odds of pacemaker implantation for new BBB (OR 2.80, 95% CI 0.61–12.87, p = 0.19), new complete RBBB (OR 3.24, 95% CI 0,57-18.36, p = 0.19), and QRS prolongation >20ms (OR 2.5, 95% CI 0.53–11.90, p = 0.25), but none of these associations were statistically significant. Consistent results were obtained from Fisher’s exact test, with p-values of 0.26, 0.21, and 0.42 respectively. Due to the predominance of atrial fibrillation, new-onset AV-block °I was analysed in a small subset of 7 patients who were in sinus rhythm before and after EVOQUE. In this subset, new onset AV block °I was significantly associated with pacemaker implantation (Fisher’s exact test, p = 0.048). Calculation of the odds ratio by logistic regression was not possible due to perfect separation as all patients with new AV block °I received a PM.

### PM implantation and electrical parameters

Details on PM implantation and electrical parameters intra- and post-operative are shown in Table 2. As previously stated 10/11 implantations followed the initial implantation strategy, whereas in one implantation the procedural approach had to be changed.

**Table 2:** PM implantation, procedural results and electrical parameters of the ventricular leads

PM-System	PM-Mode	OP-Time (min)	Dose area product (cGy*cm²)	Electrical Parameters intra-operative			Electrical Parameters post-operative		
				Impedance (Ohm)	Sensing (mV)	Threshold (V/ms)	Impedance (Ohm)	Sensing (mV)	Threshold (V/ms)
LLP	VVI	21	51	1120	8.8	0.3 /0.24	860	11.9	0.38 /0.24
LLP	VDD	45	171	682	2.9	0.5 /0.24	580	5.9	0.63 /0.24
LLP	VVI	22	81	1000	8.4	0.6 /0.24	690	7.2	0.38 /0.24
LV-P	VVI	40	83	990	4.4	0.9 /0.5	938	4.5	1 /0.4
LV-P	VVI	82	1070	700	*	1.4 /0.5	638	5.7	1.5 /0.4
LV-P	VVI	65	602	500	7	2.1 /1.5	493	9.7	2.0 /2.0
LV-P	VVI	237	1564	980	*	1.9/0.5	900	*	1.5 /1.0
LV-P	DDD	99	152	980	8	0.4/0.5	738	12.0	0.5 /0.4
LV-P	VVI	97	2148	1400	*	2.6/0.5	647	*	1.0 /1.0
LV-P	VVI	70	776	958	9	1.6/0.5	675	7.2	1.5 /1.0
RV-P	DDD	194	2860	680	18	0.5 /0.5	463	4.3**	0.5 /0.4

* no intrinsic rhythm ** Measurement of extra-systoles in complete heart block

 The choice of implanted devices and the corresponding pacing modalities reflect the high proportion of patients with permanent atrial fibrillation. Three patients were in sinus rhythm at the time of device implantation requiring a dual-chamber PM. Accordingly, one patient received a Micra AV (Medtronic) capable of VDD pacing, one patient received a dual chamber pacemaker with a CS lead and one patient received a dual chamber pacemaker with a conventional RV and RA pacing lead (Abbott). The remaining patients in permanent atrial fibrillation received single chamber PM, either leadless PM (Micra VR, Medtronic) or CS pacing leads (Abbott or Boston Scientific). All used coronary sinus lead were bipolar leads with an IS1-connector. Leadless PMs were positioned inferoseptally (1/3) or apically (2/3). The CS leads were implanted in anterior, lateral or posterolateral veins. Two procedures required prolonged OP-Time (237 min and 194 min). The first was a case of CS-lead implantation which was prolonged because of intraprocedural dislocation of the CS-lead while sheath slitting and need for re-implantation. The second case represents the patient with failure of the leadless approach as well as the of a CS-lead placement which made RV-lead placement as a bail-out strategy necessary. Temporary pacemakers were placed before permanent PM implantation in 8/11 patients. Fluoroscopy was used in all cases to guide the temporary pacing lead across the EVOQUE system. Routine post-operative device interrogations were conducted acutely within 3 days after implantation. Electrical parameters remained stable for all implanted PM systems. No peri- or postoperative complications due to PM implantation were observed in our cohort.

## Discussion

### Conduction disturbances after TTVR

High-degree AV-block requiring PM implantation is a common complication after TTVR with the EVOQUE system. In patients without a prior PM more than a third (35.5%) required a permanent PM. This rate, although higher, is comparable to the TRISCEND II trial data as well as first real world data reported by Angellotti et al. (27.8% and 18.9%) [6, 7] and underlines the significance of conduction system disorders as a relevant complication after TTVR and the need for structured PM implantation strategies.

In our cohort, the majority of PM were implanted during the index hospitalization shortly after TTVR. In total 8 out of 11 implantations were carried out within the first 7 days after TTVR. Timing of pacemaker implantation was similar in the reports by Angelotti et al., with 21/25 PM implantations in the first week and in the TRISCEND I study, where all pacemakers were implanted in the first 9 days after TTVR (n=15) [5, 7]. Risk for conduction system disorders therefore seems to be highest during the first days after the procedure. Acute onset of complete heart block intra or post-procedural is a severe complication. Specifically as precautionary placement of a temporary RV pacing wire, as routinely performed in transcatheter aortic valve replacement [11], is not feasible in the tricuspid valve replacement procedure. Placement of a temporary pacing wire over the EVOQUE system is possible but more challenging due to the valve prothesis. In our experience, access to fluoroscopy was essential in enabling temporary pacemaker placement.

The optimal time horizon for intensified surveillance post-TTVR is unclear, both as an inpatient and in the early-post-discharge ambulatory setting. This problem is aggravated by a delayed onset of conduction system disorders. In our cohort, 3 patients presented with clinically significant high-degree AV-block after discharge, one of whom presented with acute cardiogenic shock due to bradycardia. In order to address the challenge of delayed conduction system disorder after TTVR as well as allowing early discharge after the procedure, identification of patients at risk for PM implantation is of critical importance. In our cohort development of RBBB occurred in 11/31 patients after TTVR with the EVOQUE system, five of whom subsequently received a PM. Statistical analysis showed positive Odds ratios, but test results remained insignificant, most likely due to the limited sample size of our cohort. Notably 2/3 patients in our cohort with delayed onset of conduction system disorder and rehospitalisation for PM implantation developed a RBBB beforehand. Angelotti et al. identified presence of conduction disturbances at baseline as an independent predictor for PM implantation and also reported development of RBBB as the second most frequent new conduction disturbance after TTVR in 12/153 patients (following advanced AV-Block as the most frequent new conduction disturbance in 17/153 patients) [7]. Development of a RBBB could hint a subgroup at higher risk for subsequent high-degree AV-Block, mirroring the experience with LBBB after TAVR [12]. We would suggest that the development of new RBBB post-TTVR warrants an extended period of cardiac monitoring. In addition, HV interval measurement via electrophysiology studies could provide valuable risk stratification and should be evaluated in patients with conduction system disease after TTVR. Furthermore, new onset AV-block °I was associated with subsequent PM implantation in a small subset of patients in sinus rhythm in our cohort. We do interpret this finding with caution due to the small number of patients (n=7). Further studies on risk factors and ECG parameter changes after TTVR are necessary to describe ECG-dynamics after TTVR, identify patients at risk and to develop an optimal treatment approach.

### PM implantation after TTVR

To our knowledge, this is among the first valuable reports presenting data on implantation success rates of different PM systems after TTVR with the EVOQUE system. Our success rate of implanting valve sparing PM systems in 90.9% of patients and our feasibility rate of 100% compare to the report by Angelotti et al., who also achieved PM implantation in all patients. Angelotti et al. similarly to our approach reported valve sparing PM implantations in the majority of patients (19/25 patients, leadless PM in 10 and CS-lead in 9 patients) but also describe implantation of a transvalvular RV lead in 6/25 patients [7]. In TRISCEND II the type of implanted PM was recorded and again valve sparing pacing options were predominantly chosen, consisting of leadless PM, CS lead or epicardial lead placement. Right ventricular lead implantation was reported in one patient [6].

In our cohort, implantation of a leadless PM (Micra, Medtronic) was successful in 3 attempts, whereas unsuccessful in one patient because of failure to advance the delivery catheter over the bioprosthetic valve. As mentioned, successful implantations of leadless PM in patients after EVOQUE have been reported in the TRISCEND II data as well as by Angelotti et al.. While evidently possible, manoeuvring the 23 French delivery catheter (e.g. in case of Micra VR, Medtronic) over the EVOQUE’s orifice remains a critical step in the procedure which could hinder successful implantation and furthermore possibly damage or malposition the newly implanted valve. Intraprocedural imaging was not employed in our patients but could offer additional guidance during the implantation procedure, Implantation of leadless PM has also been described in case series after tricuspid valve surgery (repair/bioprothesis) or intervention (TEER) [13–15]. In particular, optimal positioning of the leadless pacemaker after bioprosthetic valve replacement remains an area for further investigation. While Fink et al. describe placement of leadless PM at the high septum in 11/12 patients after tricuspid valve surgery/intervention without complications [13], Gul et al. present a series of 40 leadless PM implantations after tricuspid valve surgery/intervention and advocate for placement at the apical septum in order to avoid problematic interaction between the PM and the valve [14]. In our experience manoeuvrability of the delivery catheter was limited in patients with an EVOQUE system, thus reaching a stable position of the leadless PM remained our primary objective. 

Placement of a CS lead is a second valve sparing pacing option. Implantation of a CS lead PM was successful in 7 patients and failed in one patient in our cohort. Altered anatomical conditions in the right atrium due to anchoring of the EVOQUE system did not prohibit or complicate access to the coronary sinus in our patients. Nevertheless, CS lead placement was not successful in one patient because of unstable positioning or unacceptable electrical parameters (high pacing thresholds, phrenic nerve stimulation) in all accessible target vessels. While pacing through leads in the coronary sinus is an established concept in Cardiac Resynchronisation Therapy (CRT) [10], antibradycardic pacing via a single coronary sinus lead carries several additional challenges. CS leads typically have no active fixation but are fixated via wedge positioning. Although lead stability is a concern in CRT, it becomes of crucial importance in isolated CS lead pacing. Moreover, sensing of the CS lead is a non-essential aspect in CRT, whereas in case of LV-lead only pacing it becomes critical for allowing demand-based pacing. In addition, while stimulation thresholds in CRT can be programmed with lower safety margins, relying on RV pacing for definite pacing, this rationale does not apply in CS-only pacing. One further technical distinction between CS leads for CRT and the use of CS pacing is the use of bipolar CS leads with IS-1 pins, that can be connected to the RV port of single dual or tri-chamber PM. Quadripolar CS leads, which are predominantly used in CRT nowadays only connect to corresponding ports in CRT-devices (IS-4) which for most manufacturers do not allow for sensing and are therefore unable to perform solo CS pacing. While these aspects make solo-CS lead placement more challenging than CS lead placement in CRT, resynchronisation is not the objective in purely bradycardia derived CS lead pacing, therefore any coronary sinus vein providing stable position and good electrical parameters is eligible, increasing the potential number of target veins in comparison to CRT. Nevertheless, apical positioning should be avoided to prevent LV dysfunction in long term pacing therapy. In summary, for CS lead placement the number of potential target veins is higher but the requirements for lead stability and electrical performance are more critical than in CRT CS lead placement.

While CS lead placement with CRT has been available for more than 20 years [16], only small case series on the feasibility of isolated CS pacing have been published [17–22]. Noheria et al. published a cohort of solo CS lead placement in 23 patients where tricuspid valve surgery prohibited standard RV lead placement. They reported successful implantation in all patients and over 95% stable lead function after a mean follow up of 5.3 years [22]. Lead migration has however remained to be a challenge since the beginning of CRT and has also been described in isolated CS pacing cohorts [16, 18, 23]. In our experience placement of a CS lead in patients after TTVR with the EVOQUE system is a feasible strategy, taking into consideration that procedural failure because of unsuitable anatomy and inacceptable electrical parameters remains a possible outcome. Addressing the risk of lead migration in isolated CS lead pacing, appropriate follow-up intervals and the use of telemonitoring should be evaluated in the long-term management of these patients.

Failure of leadless and CS lead placement occurred in one of our patients, where a switch in pacing strategy was made intraoperatively and a standard RV lead was placed through the EVOQUE system. A switch from CS lead placement to RV lead placement is straightforward as the surgical as well as the venous access are the same in both approaches. In our case the bailout to an RV lead allowed to reach a pacing solution without the need for surgical implantation of epicardial leads. No lead related recurrence of TR was noted on echocardiography postoperatively and at three months follow-up. As mentioned before, RV lead placement with an EVOQUE system has been mentioned in the TRISCEND II cohort and by Angelotti et al. without further details on valve-lead interaction. Follow-up on lead placement over a bioprosthetic valve has been reported so far only for surgical tricuspid valve replacement. In one cohort of 58 patients, placement of a RV lead placed across a surgical implanted bioprothesis was not associated with an increased rate of recurrent TR over a follow-up period of 2 years [24]. Taking into account insufficient evidence on this topic, valve sparing options are preferred in current guidelines and placement of a transvenous lead after surgical tricuspid valve replacement with bioprothesis has been deemed acceptable only in selected patients [10, 25]. Further studies on the interaction between the EVOQUE system and transvalvular RV leads will have to clarify whether this approach is an advisable alternative to leadless PM and CS lead placement in patients after TTVR.

### Clinical implications

Conduction system disorder and subsequent need for PM implantation is a frequent complication after TTVR with the EVOQUE system. Optimal treatment approaches and monitoring periods after the index procedure need to be defined, patients with signs of conduction system disorder (e.g. new onset RBBB) should be followed up closely. Placement of a temporary pacemaker lead is challenged by the bioprothesis and fluoroscopy should be utilised. Permanent PM implantation in this cohort requires specific considerations, while valve sparing options consisting of leadless PM and CS lead placement appear feasible with reasonable success rates, bailout options (change of valve sparing PM approach or change to a standard RV lead) should be available and the technical skills present in the implanting physician.

### Study limitations

We reported our first experience in PM implantation in a specific cohort. Our analysis comes from a single centre and is retrospective, and thus inherently prone to selection bias and confounding. In particular our comparison of different pacing approaches lacks randomization and long-term follow-up. Current guidelines do not specify explicit cutoff values for PM indication in patients with atrial fibrillation (e.g. maximum pause duration, minimum heart rate), thus may result in a selection bias with regard to the treating physician or centre. However, symptomatic bradycardia or a regular ventricular escape rhythm in patients with atrial fibrillation justify a pacemaker implantation. Furthermore, small sample size, short-term follow-up and non-standardized recording limit the analysis of ECG changes and identification of risk factors for PM implantation.

## Supplementary information

Below is the link to the electronic supplementary material.

Supplementary File 1 ( DOCX 26.1 KB)

## Data Availability

The data underlying this article will be shared on reasonable request to the corresponding author.
