# Supplementary material for: Strategy for pacemaker implantation following transcatheter tricuspid valve replacement: A real world single centre experience
Source: J Interv Card Electrophysiol. 2025 Dec 4;69(6):1191–9. doi: 10.1007/s10840-025-02168-8 (PMC13645897; doi:10.1007/s10840-025-02168-8)
Supplement: Supplementary file 1 [file 10840_2025_2168_MOESM1_ESM.docx]

**Strategy for Pacemaker Implantation following Transcatheter Tricuspid Valve Replacement: A Real World Single Centre Experience**

**Supplemental Material**

Supplemental Table 1: ECG Parameters in patients with new PM implantation and time to implantation:

| Pre-Evoque | |  |  |  | Post-Evoque | |  |  |  | Indication for PM | | Time to Implantation (days) |
| --- | --- | --- | --- | --- | --- | --- | --- | --- | --- | --- | --- | --- |
| Rhythm | Axis | PQ (ms) | QRS (ms) | BBB | Rhythm | Axis | PQ (ms) | QRS (ms) | BBB | |  |  |
| SR | LA | 180 | 70 | - | SR | NA | 330 | 80 |  | | HD-AVB | 6 |
| SR | LA | 180 | 85 | - | SR | SA | 280 | 110 | Incompl. LBBB | | HD-AVB | 7 |
| Afib | LA | - | 90 | - | Afib | SA | - | 140 | RBBB | | HD-AVB | 41 |
| Afib | NA | - | 90 | - | Afib | NA | - | 100 | - | | HD-AVB | 2 |
| Afib | SA | - | 100 | - | Afib | SA | - | 140 | RBBB | | HD-AVB | 3 |
| Afib | LA | - | 100 | - | Afib | LA | - | 100 | - | | HD-AVB | 6 |
| Afib | mLA | - | 115 | LAFB | Afib | mLA | - | 130 | LAFB + RBBB | | HD-AVB | 35 |
| Afib | SA | - | 85 | - | Afib | SA | - | 100 | Incompl. RBBB | | HD-AVB | 2 |
| Afib | SA | - | 85 | - | Afib | mRA | - | 120 | RBBB | | HD-AVB | 1 |
| Afib | LA | - | 100 | - | Afib | LA |  | 110 |  | | HD-AVB | 5 |
| Afib | LA | - | 95 | - | Afib | LA | - | 95 | - | | HD-AVB | 15 |

*SR, Sinus rhythm; Afib, atrial fibrillation; AVB, AV-Block; BBB, Bundle branch block; RBBB, Right bundle branch block; LAFB, Left anterior fascicular block; HG-AVB, High-degree AV-Block; NA, Normal Axis; SA, Steep Axis; LA, Left Axis; mRA, Marked Left Axis; mRA, Marked Right Axis

Supplemental Table 2: ECG parameters in patients without PM implantation:

| Pre-Evoque |  |  |  |  | Post-Evoque |  |  |  |  |
| --- | --- | --- | --- | --- | --- | --- | --- | --- | --- |
| Rhythm | Axis | PQ (ms) | QRS (ms) | BBB | Rhythm | Axis | PQ (ms) | QRS (ms) | BBB |
| Afib | LA | - | 80 | - | SR | LA | 230 | 150 | RBBB |
| Afib | NA | - | 70 | - | Afib | SA | - | 70 | - |
| Afib | LA | - | 80 | - | Afib | LA | - | 80 | - |
| SR | LA | 170 | 90 | - | SR | LA | 170 | 90 | - |
| Afib | NA | - | 80 | - | Afib | SA | - | 95 | Incompl RBBB |
| Afib | LA | - | 100 | - | Afib | LA | - | 130 | RBBB |
| Afib | SA | - | 80 | - | Afib | SA | - | 80 | - |
| Afib | LA | - | 90 |  | Afib | LA | - | 90 | - |
| Afib | LA | - | 90 | - | Afib | NA | - | 120 | RBBB |
| Afib | NA | - | 80 | - | Afib | NA | - | 100 | Incompl RBBB |
| SR | SA | 140 | 70 | - | SR | SA | 140 | 70 | - |
| Afib | NA | - | 90 | - | Afib | NA | - | 90 | - |
| Afib | LA |  | 90 |  | Afib | LA | - | 85 | - |
| SR | mLA | 210 | 115 | LAFB | SR | mLA | 250 | 115 | LAFB |
| Afib | LT | - | 75 | - | Afib | ST | - | 80 | - |
| SR | mLA | 140 | 90 | - | SR | mLA | 140 | 90 | - |
| SR | LT | 300 | 90 | - | SR | LT | 300 | 90 | - |
| Afib | S1Q3 | - | 100 | - | Afib | S1Q3 | - | 100 | - |
| Afib | NA | - | 90 | - | SR | NA | 160 | 90 | - |
| Afib | LA | - | 85 | - | Afib | LA | - | 110 | Incompl RBBB |

* SR, Sinus rhythm; Afib, atrial fibrillation; AVB, AV-Block; BBB, Bundle branch block; RBBB, Right bundle branch block; LAFB, Left anterior fascicular block; NA, Normal Axis; SA, Steep Axis; LA, Left Axis; mRA, Marked Left Axis
